# Ecological and functional consequences of coastal ocean acidification: Perspectives from the Baltic-Skagerrak System

**DOI:** 10.1007/s13280-018-1110-3

**Published:** 2018-12-01

**Authors:** Jonathan N. Havenhand, Helena L. Filipsson, Susa Niiranen, Max Troell, Anne-Sophie Crépin, Sverker Jagers, David Langlet, Simon Matti, David Turner, Monika Winder, Pierre de Wit, Leif G. Anderson

**Affiliations:** 1 Department of Marine Sciences, Tjärnö Marine Laboratory, University of Gothenburg, Strömstad, 45296 Gothenburg, Sweden; 2 Department of Geology, Lund University, 22362 Lund, Sweden; 3 Stockholm Resilience Centre, Stockholm University, Kräftriket 2B, 10691 Stockholm, Sweden; 4 Beijer Institute of Ecological Economics, Royal Swedish Academy of Science, Lilla Frescativägen 4, 10405 Stockholm, Sweden; 5 Department of Political Sciences, University of Gothenburg, Box 711, Sprängkullsgatan 19, 40530 Gothenburg, Sweden; 6 Department of Law, University of Gothenburg, Box 650, 40530 Gothenburg, Sweden; 7 Department of Political Sciences, Luleå University of Technology, 97187 Luleå, Sweden; 8 Department of Marine Sciences, University of Gothenburg, Box 461, 40530 Gothenburg, Sweden; 9 Department of Ecology, Environment and Plant Sciences, Stockholm University, 10691 Stockholm, Sweden

**Keywords:** Baltic, Ecosystem services, Eutrophication, Indirect effects, Ocean acidification, Warming

## Abstract

Ocean temperatures are rising; species are shifting poleward, and pH is falling (ocean acidification, OA). We summarise current understanding of OA in the brackish Baltic-Skagerrak System, focussing on the direct, indirect and interactive effects of OA with other anthropogenic drivers on marine biogeochemistry, organisms and ecosystems. Substantial recent advances reveal a pattern of stronger responses (positive or negative) of species than ecosystems, more positive responses at lower trophic levels and strong indirect interactions in food-webs. Common emergent themes were as follows: OA drives planktonic systems toward the microbial loop, reducing energy transfer to zooplankton and fish; and nutrient/food availability ameliorates negative impacts of OA. We identify several key areas for further research, notably the need for OA-relevant biogeochemical and ecosystem models, and understanding the ecological and evolutionary capacity of Baltic-Skagerrak ecosystems to respond to OA and other anthropogenic drivers.

## Introduction

**Fig. 1:**
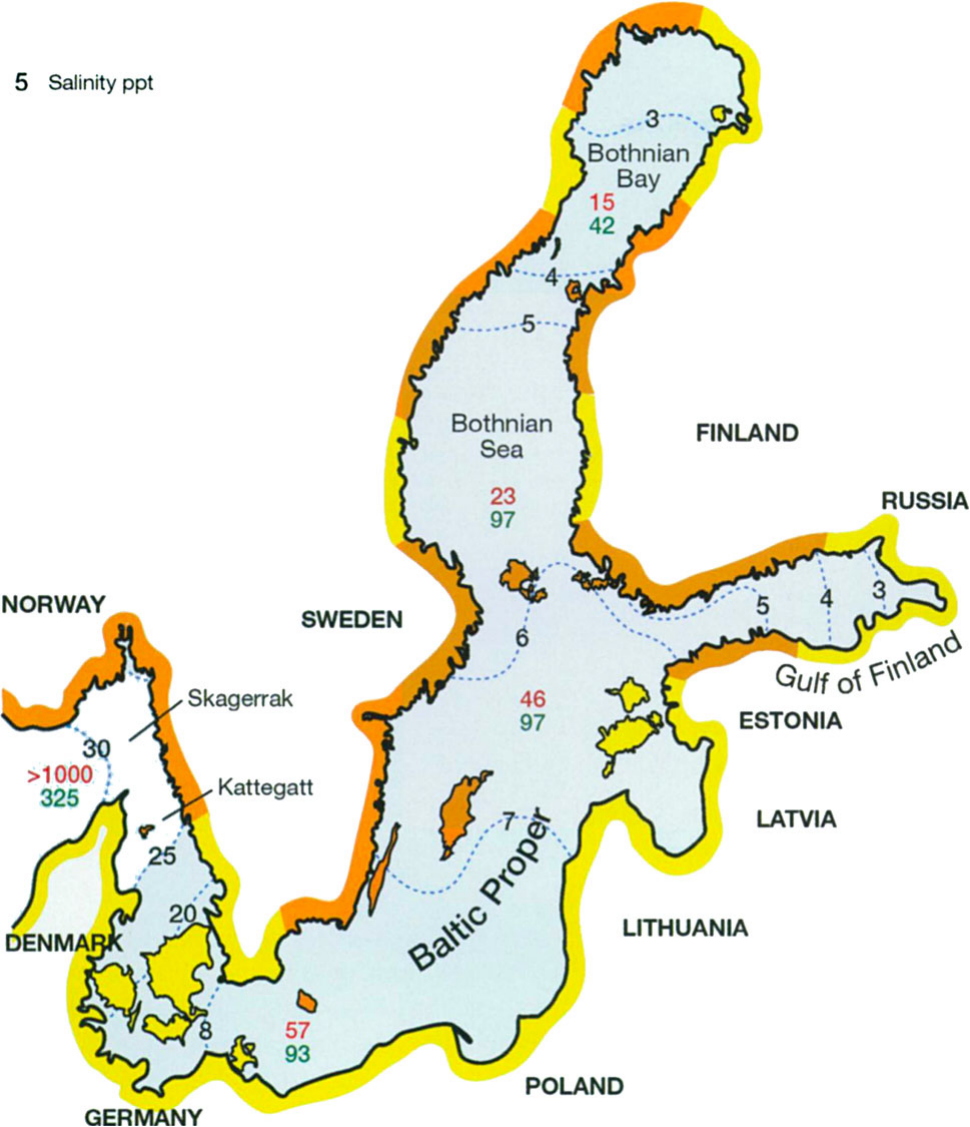
Map of the Baltic-Skagerrak System (Skagerrak, Kattegatt, Baltic Proper, Bothnian Sea, Bothnian Bay, Gulf of Finland). We use the term “Baltic-Skagerrak System” to refer to the entire region from the Skagerrak to the Bothnian Bay. Modified from Rönnbäck et al. (2007)

Globally, increasing emissions of anthropogenic carbon dioxide (CO_2_) are causing warming of the oceans, melting of sea ice, glaciers and ice sheets, and ocean acidification.^1^ For the Baltic-Skagerrak System (Fig. 1), these processes are reflected in rising sea level, increased precipitation (leading to locally reduced salinity), increased flooding, coastal erosion, and flow of organic and inorganic matter into coastal waters. All of these add to the direct and indirect effects of ocean acidification in different ways. Anthropogenic ocean acidification arises when anthropogenic emissions of CO_2_ elevate atmospheric CO_2_ concentration, resulting in elevated partial pressure of CO_2_ (pCO_2_) in the oceans and a corresponding decrease in ocean pH. But pH also varies naturally in the oceans over diurnal and seasonal timescales. This arises due to biogeochemical drivers, such as temperature, salinity, and input of terrestrial organic matter and subsequent decay, as well as through biological processes such as primary production (which increases pH) and respiration (which decreases pH). While the effects of these drivers on marine life are relatively well understood individually, we know less about the effects of combinations of drivers on single species, and little about their effects on entire coastal ecosystems, communities, and society in general—especially for ocean acidification.

Ocean acidification (hereafter, ‘OA’) is recognised in the United Nations’ Sustainable Development Goal 14.3 “*Minimize and address the impacts of ocean acidification…”*, and has been identified as a cause of substantial impacts on marine ecosystems (Gattuso et al. 2015; Riebesell and Gattuso 2015). Although OA is a global issue caused by rising atmospheric CO_2_ concentrations (Doney et al. 2009), the degree and effects of OA are geographically heterogeneous due to regional differences in air:sea fluxes and ocean chemistry (Steffen et al. 2015). Multiple recent reviews of OA have concluded that effects on species are generally negative, but that responses vary markedly among species (e.g. Kroeker et al. 2013), notably between field and the laboratory (e.g. Wahl et al. 2018), and that OA effects can be strongly modified by interactions with other drivers (Gunderson et al. 2016) and species (Kroeker et al. 2017). Much of this recent work highlights the importance of non-linear processes, especially when multiple environmental drivers interact (e.g. Albright et al. 2016a; Mostofa et al. 2016; Kroeker et al. 2017). For example, Gao et al. (2018) found that the interactive effects of OA and ocean warming on phytoplankton physiology could be synergistic, neutral, or antagonistic depending on species and prevailing environmental conditions. Equivalent responses have been reported at higher trophic levels (Harvey et al. 2013). At a broader scale, OA impacts on marine ecosystems have the potential to affect a wide range of ecosystem services (ESs). Although a general picture of OA effects on ESs is yet to emerge (Falkenberg and Tubb 2017), some specific OA-related dis-benefits have already been identified (e.g. oysters, Lemasson et al. 2017). Addressing these OA impacts directly may be difficult, however, manipulating non-OA drivers affecting ecosystem resilience or adaptation has been suggested as a viable management policy option to mitigate OA effects (Albright et al. 2016b).

The geological record provides an important environmental archive of past OA events and how past marine ecosystems have responded to changes in pH and ocean biogeochemistry (e.g. Harnik et al. 2012; Hönisch et al. 2012). The most recent global event occurred during the last deglaciation, when atmospheric CO_2_ increased 30% (from 189 to 265 μatm), leading to a ~ 0.15 unit drop in sea surface pH in the open ocean (e.g. Hönisch and Hemming, 2005). This change corresponded to a decrease of 0.002 units per 100 years, which is much slower than current rates (Zeebe et al. 2016). The coastal seas of the Baltic-Skagerrak System were subjected to considerable environmental changes during the deglaciation and disentangling the potential effect of a regional change in pH (and its magnitude) remains to be done.

The Baltic-Skagerrak System comprises one of the world’s largest permanent salinity gradients (Fig. 1) from the high salinity Skagerrak shores (salinity ~ 30)^2^ to the shallow, almost freshwater archipelagos of the northern Bothnian Bay (salinity ~ 3). This gradient not only contains multiple different ecosystems, but also creates differences in seawater chemistry that alter the process, and effects, of OA. Hence ocean acidification will impact various parts of the system very differently.

In addition to drivers^3^ such as OA and warming that are changing marine systems on a global scale, the Baltic-Skagerrak System is also subject to local pressures from eutrophication, freshening, and pollution, resulting from agriculture, tourism, fishing, aquaculture, etc. Local differences in the strength and timing of these drivers, and the natural heterogeneity of coastal seas, create far greater variability and change than that seen in the open ocean. Coastal marine ecosystems of the Baltic have changed markedly during the past 40 years, which is partly attributable to local and regional warming and freshening (Olsson et al. 2012), as well as eutrophication (Olsson et al. 2015). The role of OA in this context is poorly known, not least because most biologists were unaware of its importance until relatively recently (Gattuso and Hansson 2011).

Here we (i) outline the current state of knowledge related to OA in the Baltic-Skagerrak System, as an example of OA-related processes in a brackish coastal sea; (ii) identify knowledge gaps; and (iii) suggest priorities for future research. We focus on the mechanisms that underlie OA, its interactions with other coastal drivers, and its impacts on biogeochemical processes, marine species, and ecosystems. Unless stated otherwise, we consider the biological consequences of “near-future” OA (i.e. pCO_2_ levels up to ~ 1300 µatm, van Vuuren et al. 2011). Information summarised here was obtained from ISI Web of Science and EconLit databases using the search terms “ocean acidification” or “pCO_2_”, together with “Baltic, Kattegat, Skagerrak or Finland”. Additional examples, including non-OA examples, were searched for on an ad hoc basis. A companion manuscript (Jagers et al. 2018) addresses the socio-economic background to, and prospective societal solutions for, OA in Swedish coastal seas. Throughout, we use the term “Baltic-Skagerrak System” to refer to all waters from the Skagerrak to the Bothnian Bay, and the relevant regional terms for the different components of the Baltic system (Fig. 1).

## Biogeochemical basis of ocean acidification in the Baltic-Skagerrak System

**Fig. 2:**
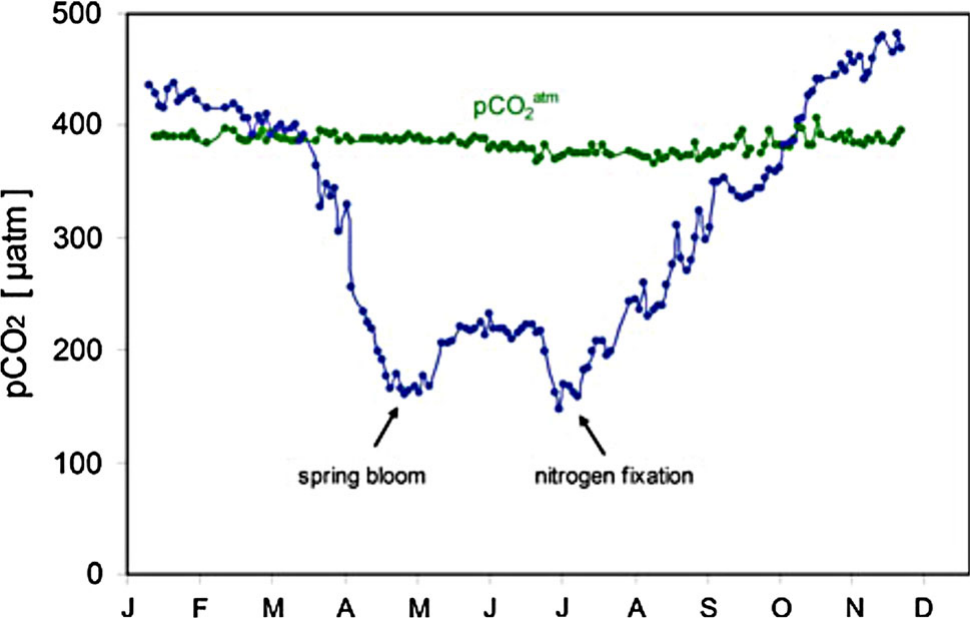
Seasonal changes in phytoplankton productivity absorb dissolved CO_2_ in the water (blue line) during spring and summer, creating “draw-down” relative to the level of CO_2_ in the overlying atmosphere (green line). Data from central Baltic Sea, taken from Schneider et al. (2015)

The surface ocean continuously exchanges CO_2_ with the atmosphere. When atmospheric partial pressure of CO_2_ (pCO_2_) is greater than that in the oceans, there is a flux of CO_2_ from the atmosphere into the oceans (and vice versa). This flux is influenced by primary production—which reduces seawater pCO_2_ in the summer, and by decomposition and biomineralisation—which cause the release of CO_2_ in the winter. These processes create a seasonal cycle of pCO_2_ both in the atmosphere and the ocean. However, as the atmosphere mixes much faster than the ocean, the atmospheric signal is diluted and far less pronounced: for instance the seasonal amplitude in atmospheric pCO_2_ at the latitude of Sweden is ~ 10 µatm, while that in the coastal ocean can be several hundred µatm (Fig. 2).

**Fig. 3:**
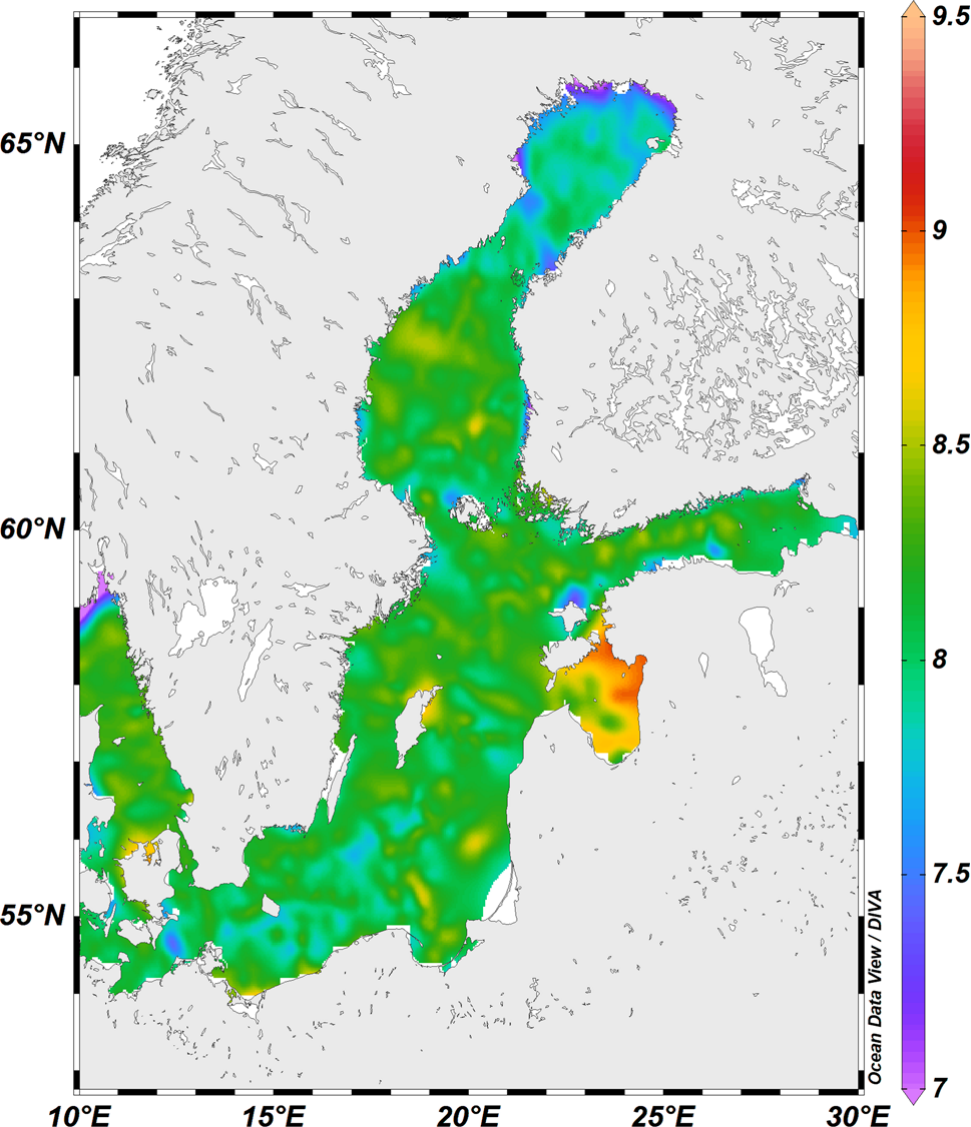
Long-term mean surface water (0–20 m) pH in the Baltic-Skagerrak System, © Adam Ulfsbo. Data are interpolation of historical records collected during all seasons between 1911 and 2003, and therefore only show general patterns in pH distribution. For more information, including station positions, see Hjalmarsson et al. (2008)

Since the onset of the industrial revolution, seasonally averaged global atmospheric pCO_2_ has risen from around 280 µatm to > 400 µatm today, and consequently the average pCO_2_ of the surface ocean has also increased (Bates et al. 2012). Seasonal fluctuations in pCO_2_ are superimposed on this mean increase, leading to greater extremes of pCO_2_. Increasing atmospheric pCO_2_ reduces ocean pH, but the extent of pH reduction depends not only on the pCO_2_ itself, but also on other properties of the seawater, notably the existing pH, the buffering capacity (the “alkalinity”) of the seawater, and to a small extent the temperature (see Box 1). The chemical buffer capacity, i.e. the change in pH for a given change in pCO_2_, is determined by the total concentration of all the bases in the seawater, measured as total alkalinity. In most of the world’s coastal oceans, alkalinity is broadly correlated with salinity, and therefore buffer capacity typically decreases with decreasing salinity. However, in the different basins of the Baltic-Skagerrak System, this alkalinity:salinity relationship is complicated by large differences in local geology that influence alkalinity independently (Omstedt et al. 2009; see Box 2). This results in the different low-salinity basins of the Baltic-Skagerrak System having different pHs despite having similar salinities; i.e. low pH in the northern Bothnian Bay and inner Gulf of Finland due to input of low alkalinity river runoff, and high pH in the Gulf of Riga resulting from inflow of high alkalinity runoff (Fig. 3).

**Table:** 

**Box 1:** Atmospheric CO_2_ and the acidification of seawater	
Unlike the other atmospheric gases, when CO_2_ dissolves in seawater it reacts chemically with the water: ${\text{CO}}_{ 2} \left( {\text{aq}} \right) \, + {\text{ H}}_{ 2} {\text{O}} \,\leftrightarrows \,{\text{H}}_{ 2} {\text{CO}}_{ 3}\, \leftrightarrows\, {\text{H}}^{ + } + {\text{ HCO}}_{ 3}^{-}\, \leftrightarrows\, {\text{2H}}^{ + } + {\text{ CO}}_{ 3}^{{ 2{-}}}$ The pH of the seawater determines which of these chemical species dominate. Higher pH drives the system farther to the right. At seawater pH’s typical of Swedish coastal waters (7.5–8.5) the carbonate system is dominated by the HCO_3_^–^ terms, and hence dissolving CO_2_ in seawater leads to an increase in proton (H^+^) concentration, and hence increased acidity. Dissolved inorganic carbon (DIC) buffers the dissolution of CO_2_ in seawater by the CO_3_^2–^ ion reacting with the CO_2_. Because DIC and alkalinity decline from the Swedish west-coast, through the Baltic Proper and into the Bothnian Bay, the effects of increasing pCO_2_ are greater in the Baltic than on the west coast (see main text).

**Table:** 

**Box 2:** Salinity, alkalinity and pH in the Baltic	
In the Baltic, sea surface salinity is determined by the combination of runoff of freshwater together with limited exchange of seawater with the North Sea. In those parts of the Baltic system most distant from the North Sea (Bothnian Bay and eastern Gulf of Finland), the salinity is below 3, rises to around 7 in the Baltic Proper, and then rises rapidly from ~ 8 in the southern Danish straits to ~ 15 in the Kattegat just a hundred km or so to the north. Salinity continues to rise to the north and west, reaching 30 in the western Skagerrak (Fig. 1). The alkalinity of river runoff varies depending on the geology of the drainage basin. The northern Bothnian Bay, which is largely surrounded by granite bedrock, has relatively low alkalinity and pH because the alkalinity of the runoff is low. In contrast, the limestone bedrock that characterises the watersheds flowing into the Gulf of Riga has very high alkalinity, and thus pH in the Gulf of Riga is much higher (Fig. 3). Recent work shows increased weathering of bedrock, increases alkalinity and offsets the effects of ocean acidification (Müller et al. 2016).

**Fig. 4:**
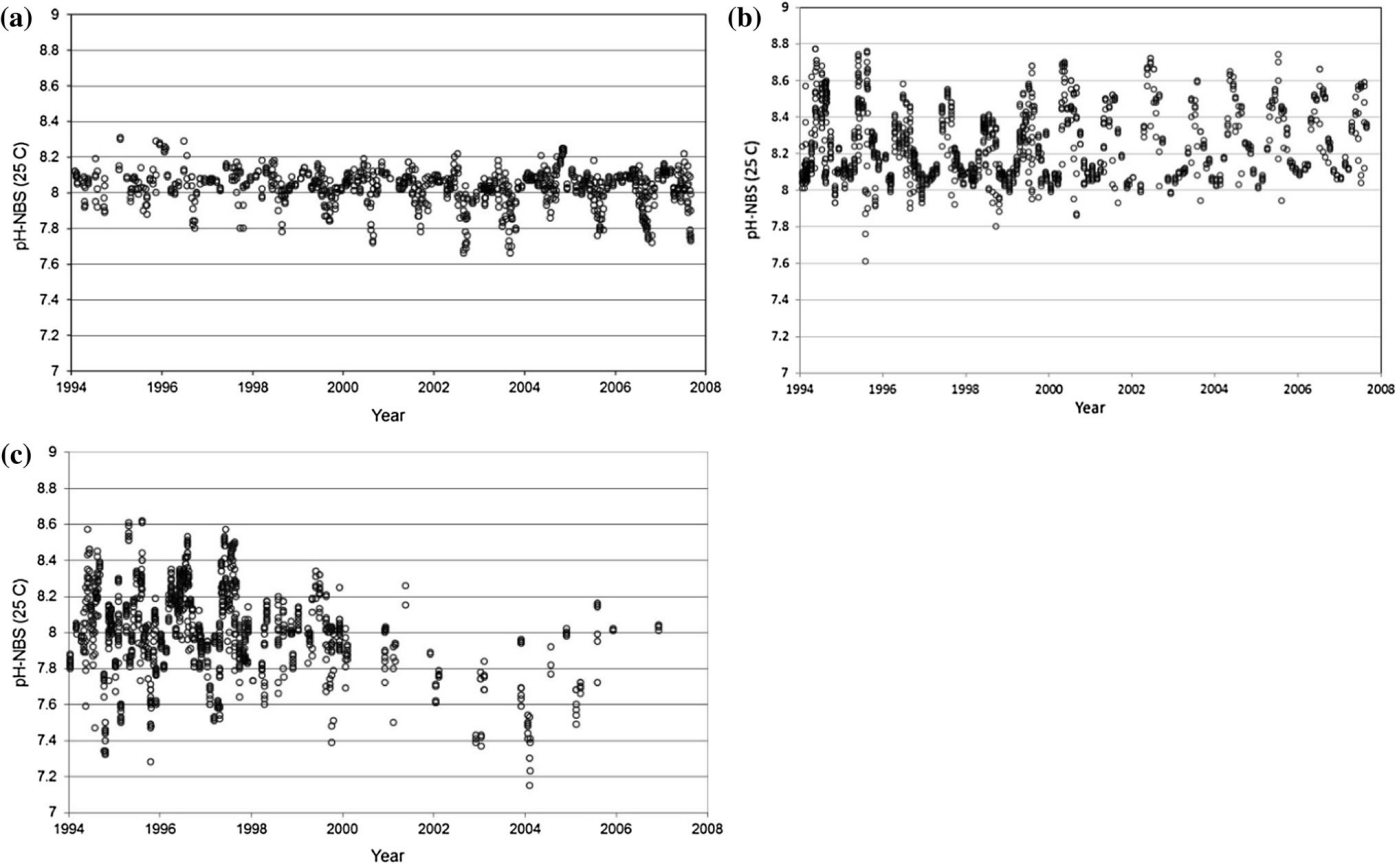
Time series of pH in the top 20 m of the water column showing seasonal fluctuations in the open Kattegat (**a**), Baltic Proper (**b**) and Bothnian Bay (**c**). Note that the observational frequency decreased substantially after 2000 in the Bothnian Sea (Data on pH_NBS_ scale, measured at 25 °C from SMHI: http://sharkdata.se/about/)

Differences in alkalinity combine with differences in the extent of primary production to cause greater seasonal variability in pH in parts of the Baltic compared to the Kattegatt/Skagerrak (Fig. 4). This effect is amplified by the nutrient-rich conditions in the Baltic that cause a stronger “draw-down” of CO_2_ during the productive season, which results in higher pH during the summer (Fig. 4b). These data are for the open Baltic and Kattegat: in shallow coastal embayments, the processes contributing to this variation generate even stronger pH variation (e.g. Saderne et al. 2013; Wallace et al. 2014; Carstensen et al. 2018).

Seasonal changes in CO_2_ uptake and release also have implications for other important chemical processes in coastal seas. Photosynthesis by micro- and macroalgae, and marine plants, consumes “acidic” CO_2_ and hydrogen ions (H^+^) (see Box 3). This causes pH to increase in summer while the decomposition of organic matter, produces hydrogen ions and lowers pH, reversing this process in winter (Fig. 2). Primary production occurs close to the surface in the surface-lit (photic) zone, but much of the decomposition occurs after this production has died and settled to the seafloor, i.e. deep in the water column or at the sediment surface. In waters with limited exchange, this can completely deplete the available oxygen near the seabed, resulting in bottom waters with very low oxygen (“hypoxic”, i.e. < 30% O_2_ saturation), or no oxygen (“anoxic”). This influences seawater chemistry resulting in increased solubility of some metals (notably manganese and iron), and in some cases the production of elemental sulphur, all of which influence seawater pH (see Box 3).

**Table:** 

**Fig. 5:**
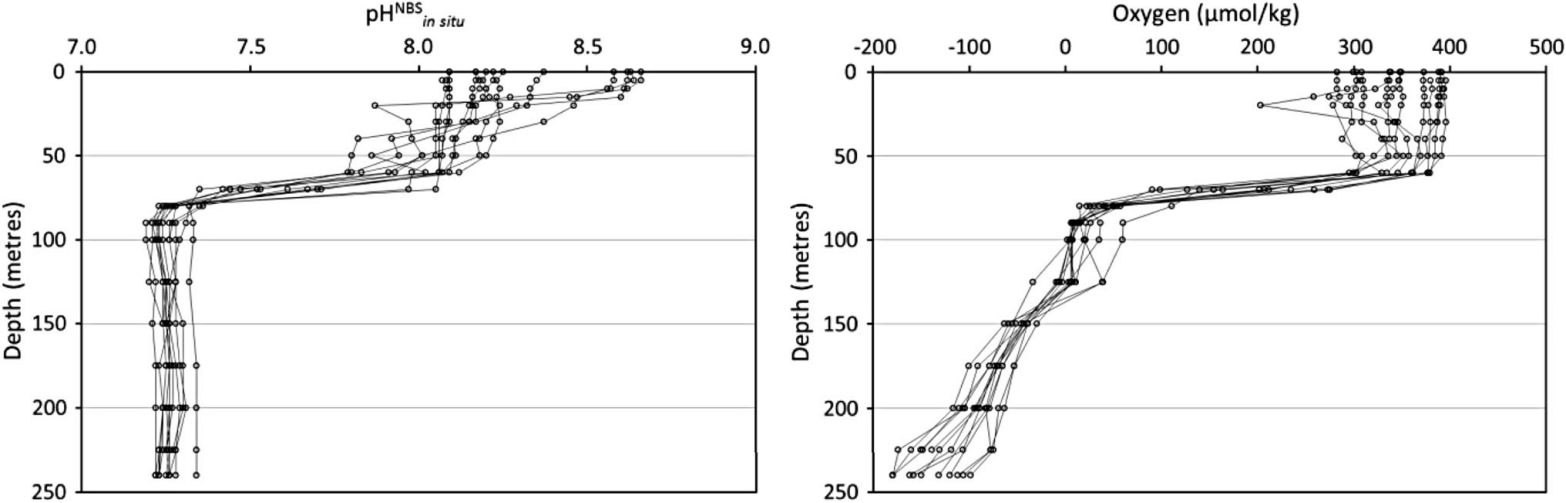
Profiles of pH and oxygen in the Gotland Basin for all months during 2008 (after Ulfsbo et al. 2011). Note that negative oxygen concentrations represent sulphide

**Box 3:** Chemical consequences of ocean acidification in the Baltic-Skagerrak System	
In the surface mixed layer of the oceans, the photosynthetic capture of light energy to combine CO_2_, macro-nutrients (such as nitrate and phosphate), and micro-nutrients in the form of trace metals (Me^2+^, such as iron(II)) to create organic matter and oxygen can be formulated as $140{\text{ CO}}_{2} + {\text{ 16 H}}^{ + } + {\text{ 16 NO}}_{3}^{ - } + {\text{ HPO}}_{4}^{ 2- } + {\text{ Me}}^{{2 + }} + {\text{ 123 H}}_{2} {\text{O}} \to ({\text{CH}}_{2} {\text{O}})_{{91}} ({\text{CH}}_{2} )_{{16}} ({\text{NHCH}}_{2} {\text{CO}})_{{16}} ({\text{CHPO}}_{4} {\text{Me}}){\mkern 1mu} + {\text{ 172 O}}_{2}$ (1) Decomposition of sedimenting organic matter in deeper water runs in the opposite direction, releasing CO_2_ and H^+^. In the following formulation, this CO_2_ release is illustrated by balancing it to the bicarbonate ion, HCO_3_^−^, the dominating form of dissolved inorganic carbon at typical seawater pH (Box 1). $({\text{CH}}_{2} {\text{O}})_{{91}} ({\text{CH}}_{2} )_{{16}} ({\text{NHCH}}_{2} {\text{CO}})_{{16}} ({\text{CHPO}}_{4} {\text{Me}}){\mkern 1mu} + {\text{ 172 O}}_{2} + {\text{ 17 H}}_{2} {\text{O}} \to 140{\text{ HCO}}_{3}^{ - } + {\text{ 156 H}}^{ + } + {\text{ 16 NO}}_{3}^{ - } + {\text{ HPO}}_{4}^{ 2- } + {\text{ Me}}^{{2 + }}$ (2) Thus decomposition produces hydrogen ions, and hence lowers pH. This reaction normally occurs deep in the water column or at the sediment surface. In waters with limited exchange, the decomposition process (reaction 2) can sometimes completely deplete the available oxygen, resulting in strongly hypoxic or anoxic bottom water. Under these circumstances, other “electron acceptors” are needed to replace oxygen in the decomposition process. The most energetically favourable electron acceptor after oxygen is nitrate, and hence in hypoxic and anoxic areas, decomposition leads to denitrification: $({\text{CH}}_{2} {\text{O}})_{{91}} ({\text{CH}}_{2} )_{{16}} ({\text{NHCH}}_{2} {\text{CO}})_{{16}} {\text{C}}({\text{MeHPO}}_{4} ){\mkern 1mu} + {\text{ 112 NO}}_{3}^{ - } {\text{ }} \to 140{\text{ HCO}}_{3}^{ - } + {\text{ 12 H}}^{ + } + {\text{ 56 N}}_{2} + {\text{ 16 NH}}_{4}^{ + } + {\text{ HPO}}_{4}^{ 2- } + {\text{ Me}}^{ 2+ } + {\text{ 23 H}}_{2} {\text{O}}$ (3) When comparing reactions (2) and (3), it can be seen that denitrification generates far fewer hydrogen ions per bicarbonate ion produced. If decomposition proceeds to deplete all the nitrate, then other electron acceptors step in. In seawater, these are (in order) manganese(IV), iron(III) and sulphate. When these are used as electron acceptors the following reactions (4–6) occur (here organic matter is simplified to “carbohydrates”; CH_2_O_(org)_): ${\text{CH}}_{2} {\text{O}}_{{\left( {{\text{org}}} \right)}} + {\text{ 2 Mn}}\left( {{\text{IV}}} \right){\text{O}}_{2} + {\text{ 3 H}}^{ + } {\text{ }} \to {\text{HCO}}_{3}^{ - } + {\text{ 2 H}}_{2} {\text{O }} + {\text{ 2 Mn}}\left( {{\text{II}}} \right)^{{2 + }}$ (4) ${\text{CH}}_{2} {\text{O}}_{{\left( {{\text{org}}} \right)}} + {\text{ 4 Fe}}\left( {{\text{III}}} \right){\text{OOH }} + {\text{ 7 H}}^{ + } {\text{ }} \to {\text{HCO}}_{3}^{ - } + {\text{ 6 H}}_{2} {\text{O }} + {\text{ 4 Fe}}\left( {{\text{II}}} \right)^{{2 + }}$ (5) ${\text{CH}}_{2} {\text{O}}_{{\left( {{\text{org}}} \right)}} + {\mkern 1mu} 0.5{\text{ SO}}_{4}^{{2 - }} {\text{ }} \to {\text{HCO}}_{3}^{ - } + {\mkern 1mu} 0.5{\text{ H}}^{ + } + {\mkern 1mu} 0.5{\text{ HS}}^{ - }$(6) These reactions have very different impacts on pH as both manganese and iron reduction consume H^+^, whereas sulphate reduction produces H^+^. An important consequence of this is that the sulphide bottom-water that occurs in the Baltic Proper has close to constant pH, even if the sulphide concentration increases with depth (Fig. 5). Note that temperature affects these biogeochemical reactions as well as the solubility of gases, which results in lower pH (more CO_2_) in colder waters in equilibrium with the atmosphere. These biochemical processes also occur in the sediment, especially in surface layers where “bioturbation” by the fauna causes mixing of interstitial water with deep waters in the water column. When anoxic water meets oxic water the reduced chemical species are oxidised in reactions that also involve hydrogen ions. For example, when iron(II) is oxidised H^+^ is produced: ${\text{Fe}}({\text{II}})^{{2 + }} + {\text{ 2 H}}_{2} {\text{O}} \to {\text{Fe}}\left( {{\text{III}}} \right){\text{OOH }} + {\text{ 2 H}}^{ + }$ (7) But when hydrogen sulphide is oxidised to elemental sulphur H^+^ is consumed: ${\text{HS}}^{ - } + {\mkern 1mu} 0.5{\text{ O}}_{2} + {\text{ H}}^{ + } {\text{ }} \to {\text{H}}_{2} {\text{O }} + {\text{ S}}^{0}$ (8) Or if iron sulphide precipitates H^+^ is produced: ${\text{Fe}}\left( {{\text{II}}} \right)^{{2 + }} + {\text{ HS}}^{ - } {\text{ }} \to {\text{FeS}}\left( {\text{s}} \right){\mkern 1mu} + {\text{ H}}^{ + }$(9)

In the Baltic-Skagerrak System, nutrient emissions from waste-water treatment and agricultural runoff have caused eutrophication, such that areas of hypoxia/anoxia have increased (Conley et al. 2009, 2011). Anoxia amplifies the effects of eutrophication through chemical reactions between iron (III) and phosphate, which in oxic sediments form precipitations that trap phosphate, but which is dissolved and released when the sediment becomes anoxic and iron(III) is reduced to iron(II). Thus, in anoxic waters phosphate is released back to the water column where, in time, it is mixed up to the surface where it stimulates primary productivity, leading to higher summer pH, as illustrated in the Baltic Proper (Fig. 4).

In addition to influencing alkalinity, river runoff also contains dissolved and particulate organic matter, some of which decays by microbial activity, adding to local deoxygenation and ocean acidification. The dissolved fraction has further impact on pH as these dissolved molecules contain carboxyl groups (organic acids) and thus can reduce pH further. Although uncertainty is large, climate model projections for the coming century show increased precipitation over northern Scandinavia, particularly in the winter months (Christiansen et al. 2015), which implies increased flux of terrestrial organic matter to the coastal seas, potentially adding to OA and deoxygenation.

**Fig. 6:**
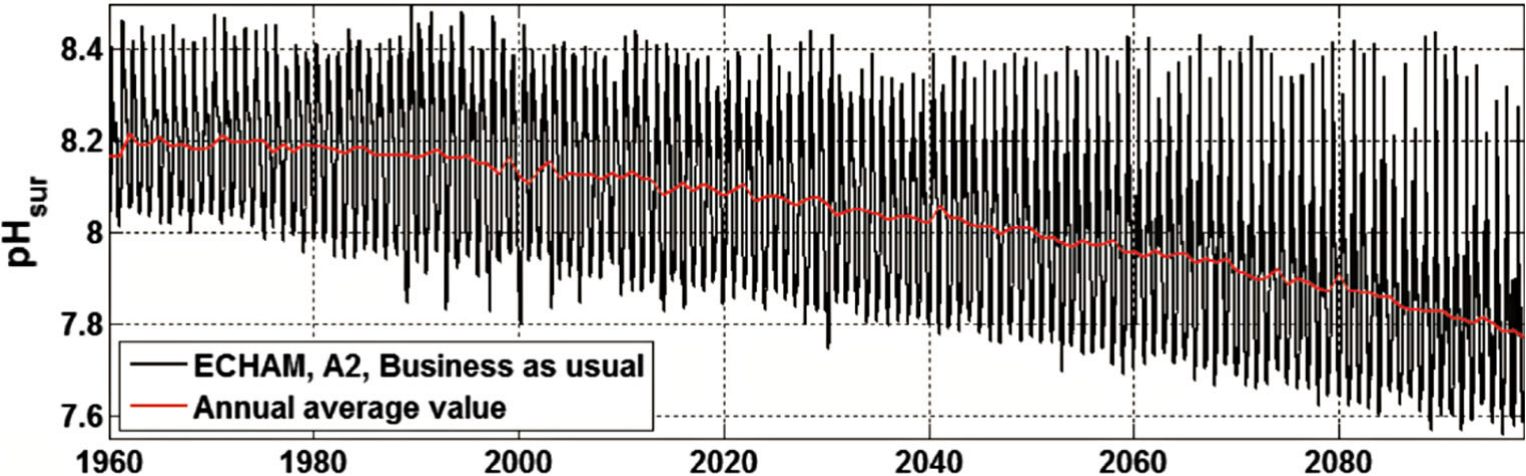
Daily pH values for surface water in the Eastern Gotland Basin projected from the ECHAM global climate model and the SRES A2 “business as usual” scenario. Note that due to increased nutrient load that stimulates photosynthetic uptake of CO_2_ from surface waters, summer maximum pH remains mostly constant until ~ 2090, whereas winter minimum pH declines almost linearly throughout the modelled period. (From Omstedt et al. 2012)

Despite many years of monitoring data, seasonal variation and methodological issues preclude reliable detection of long-term trends in measurements of seawater pH in Baltic-Skagerrak System. Recent modelling, however, projects that the combination of this variation with increasing anthropogenic atmospheric CO_2_ will lead to greater pH variation and lower pH minima in the surface waters (Fig. 6). Importantly, although projected average pH in the scenario plotted in Fig. 6 reaches 7.8 in the last decade of this century, winter minimum pH already begins to fall below this value in the year 2040. Thus, for species that are sensitive to low pH, the seasonal effects of acidification may be felt far sooner than would be expected from the projected mean annual pH.

## Direct effects of ocean acidification

Ocean acidification impacts the species of the Baltic-Skagerrak System directly, interactively (i.e. in combination with other drivers), and indirectly through competitive and trophic interactions in the ecosystem. In this section, we consider the direct effects of OA on species. The interactive and indirect effects of OA are considered in subsequent sections.

Responses of plankton in the Baltic-Skagerrak System to OA in the form of elevated pCO_2_ are highly variable, and often context dependent. This applies both among species/OTUs (operational taxonomic units), but also at the community level (Table 1). At the species/OTU level, bacterioplankton abundance typically shows no (or small) responses to OA (e.g. Baltic Proper, Lindh et al. 2013), but at the community level indirect responses can manifest as shifts in community composition, which can be linked to shifts in the phytoplankton community (western Baltic, Bergen et al. 2016; Gulf of Finland, Hornick et al. 2017). Responses of phytoplankton tend to be more variable. Cyanobacterial species in different functional groups display positive, negative, or no, responses to elevated pCO_2_ (western Baltic, Czerny et al. 2009; Eichner et al. 2014), and responses of spring-bloom diatoms and dinoflagellates also differ. For example, in the Skagerrak, growth rates of diatoms increased under OA (Kremp et al. 2012), but not growth of a toxic dinoflagellate (although toxin production increased, Kremp et al. 2012). Calcification and growth of coccolithophorids under OA varies among clones (multiple locations, Langer et al. 2009) and species (Meyer and Riebesell, 2015), illustrating the difficulties that intra- and interspecific variation create for making generalisations (Eichner et al. 2014; note that coccolithophorids are largely restricted to the higher salinity Kattegat and Skagerrak). At the community level, mesocosm studies show OA can influence phytoplankton community structure, with subtle shifts in dominant taxa such as diatoms, cryptophytes, and cyanobacteria, but stronger shifts in sub-dominant taxa (western Baltic, Sommer et al. 2015; Gulf of Finland, Paul et al. 2016a, b). Overall phytoplankton productivity seems to increase under OA, although there is also a strong seasonal component to this response (Skagerrak, Eberlein et al. 2017).

**Table 1:**
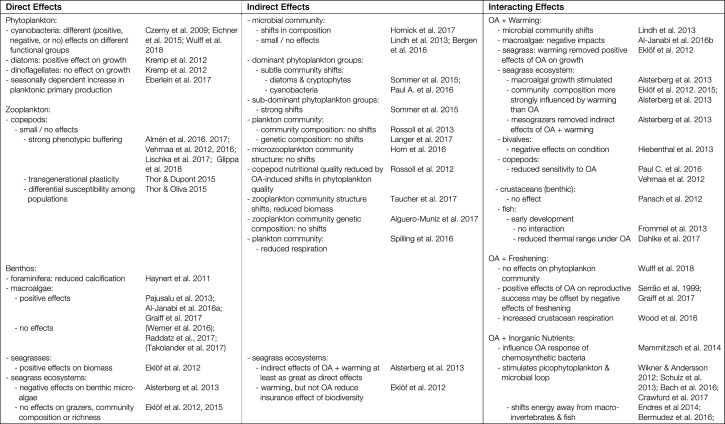

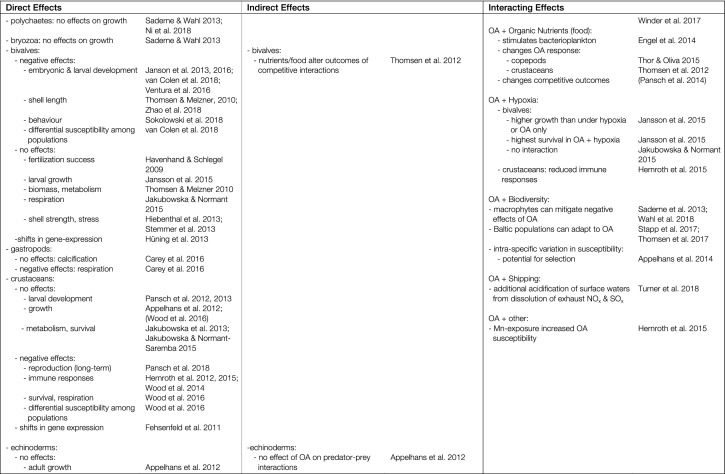

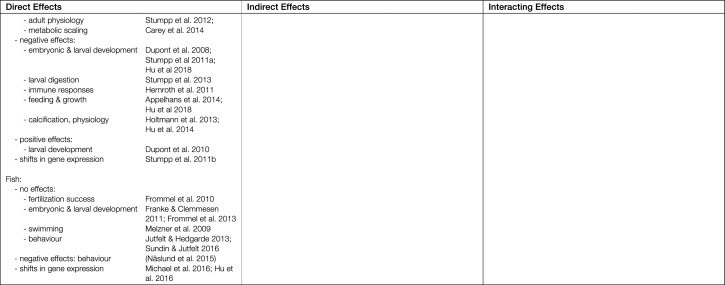
Summary of observed direct and indirect biological effects of ocean acidification (OA) and interactions with other key anthropogenic drivers in the Baltic-Skagerrak System [Effects and interactions relevant to the Baltic-Skagerrak System, but for which supporting examples come from other regions, are discussed in the main text. References in parentheses show mixed responses]

Among benthic macrophytes, macroalgae show negative, no, or positive, responses to increasing pCO_2_ (reviewed by Takolander et al. 2017). Elevated pCO_2_ stimulated growth of filamentous “opportunistic” green- and red-algae (eastern Baltic, Pajusalu et al. 2013), and of the brown alga *Fucus*, from the western Baltic and Baltic Proper (Al-Janabi et al. 2016a), and had small positive effects on fertilisation success in *Fucus* (western Baltic, Graiff et al. 2017). Other experiments with *Fucus* found no (or minor) effects of OA (Pajusalu et al. 2013; western Baltic, Werner et al. 2016; Raddatz et al. 2017). In contrast, seagrasses generally respond more uniformly, and positively, to elevated pCO_2_, although reported effects are relatively small (Skagerrak, Eklöf et al. 2012; Alsterberg et al. 2013; Portugal, Repolho et al. 2017). In cases where OA has been investigated in combination with warming, responses to OA are frequently overshadowed by the interactive effects of warming (see below).

Studies of zooplankton in the Baltic-Skagerrak System have focused on copepods, which show a high degree of phenotypic buffering to OA in a variety of traits such as body size, reproduction, and behaviour (e.g. Vehmaa et al. 2012, 2016; Almén et al. 2016, 2017; Lischka et al. 2017). Some of these responses have been shown to be enhanced by trans-generational inheritance of plasticity, whereby parental exposure to OA generates more OA-tolerant offspring (Kattegatt, Thor and Dupont, 2015; Baltic Proper, Vehmaa et al. 2016). This plasticity has limits, however, and high pCO_2_ still had negative effects (*Acartia*, 1230 µatm CO_2_, Vehmaa et al. 2016). Importantly, susceptibility of copepods to OA has been shown to vary with location and environmental history (Spitzbergen vs. Skagerrak, Thor and Oliva, 2015). This work also showed that different energetic functions (e.g. ingestion and respiration) can respond differently to OA, yielding non-linear responses at the whole-organism level (Thor and Oliva, 2015). Data for OA responses of gelatinous plankton in the Baltic-Skagerrak System are lacking, however, studies of relevant species from nearby regions indicate they are generally tolerant of OA (*Aurelia*, North Sea, Algueró-Muñiz et al. 2016), or respond positively (*Oikopleura*, North Sea, Winder et al. 2017; Bouquet et al. 2018). In summary, these data suggest that the impacts of OA on zooplankton species in the Baltic-Skagerrak System are likely to be small. Interactions among species and effects on the plankton community as a whole are considered in the following section.

In contrast to zooplankton, benthic animals in the Baltic-Skagerrak System generally respond either negatively or neutrally to OA (to date there is one report of a positive response; seastar, Skagerrak, Dupont et al. 2010). Negative responses include reduced embryonic and larval survival (brittle stars, Skagerrak, Dupont et al. 2008; bivalves, Baltic, Jansson et al. 2016; Skagerrak, Ventura et al. 2016; van Colen et al. 2018), delayed larval development (sea urchins, Skagerrak, Stumpp et al. 2011a), and immune suppression (crustaceans, Skagerrak, Wood et al. 2014; Hernroth et al. 2015; see Table 1 for full list). Reports of no, or minor, responses to OA include effects on fertilisation success (bivalves, Skagerrak, Havenhand and Schlegel 2009), adult growth (bivalves, Baltic, Thomsen and Melzner 2010), larval development (crustaceans, Skagerrak, Baltic, Pansch et al. 2012, 2013), and metabolic scaling (echinoderms, Skagerrak, Carey et al. 2014; see Table 1 for full list). Notably, bivalves from parts of the western Baltic that routinely experience strong upwelling/low pH events appear to be tolerant of extreme levels of OA (Thomsen and Melzner 2010; Thomsen et al. 2013), indicating that here too environmental history may be an important determinant of tolerance (Thomsen et al. 2017). As noted earlier for copepods, trans-generational acclimation to OA has also been reported for benthic invertebrates (e.g. Dupont et al. 2013; Hu et al. 2018), and here too, prior environmental history appears to influence tolerance (Hu et al. 2018). Dissecting the effects of trans-generational plasticity from other forms of plasticity is complex and more, targeted experimentation is required before generalisations can be made on the potential for trans-generational plasticity in benthic animals (Torda et al. 2017).

Two bivalve species in particular, *Mytilus trossulus/edulis* and *Macoma baltica*, are key components of Baltic-Skagerrak ecosystems, and fundamental to benthic ecosystem functioning in the region (Niiranen et al. 2013). In both cases, these species show few or no responses to OA: *Mytilus* from the Baltic-Skagerrak System tend to be highly insensitive to OA (Thomsen and Melzner 2010; Thomsen et al. 2010, 2013; Jakubowska and Normant 2015; Ventura et al. 2016), and this response appears to be heritable (Stapp et al. 2017; Thomsen et al. 2017). Although larvae of *Macoma baltica* from the eastern Baltic are negatively impacted by OA (Jansson et al. 2013, 2016; unlike the adjacent North Sea, van Colen et al. 2018), juvenile survival was positively impacted by OA (in combination with hypoxia, Jansson et al. 2015), and adult *Macoma* appear to be unaffected, even by very low pH (southern Baltic, Jakubowska and Normant-Saremba 2015). Thus, it seems unlikely that OA will have substantive impacts on these key bivalves—and hence on the primary benthic component of benthic-pelagic coupling—in the Baltic-Skagerrak system.

Commercially and ecologically important fish species in the Baltic-Skagerrak System show generally, but not exclusively, negative direct responses to OA. Growth of larval halibut, herring, and cod were all negatively impacted by OA (Baltic, Franke and Clemmesen 2011; Frommel et al. 2013; Skagerrak, Gräns et al. 2014; Stiasny et al. 2016), but OA had no effects on sperm motility or swimming performance of adult cod (Baltic, Melzner et al. 2009; Frommel et al. 2010). Incorporating the effects of OA in stock recruitment models led to a projected reduction in recruitment of western Baltic cod stocks by 90% (Stiasny et al. 2016). Work on non-commercial species has shown the potential for trans-generational effects that can increase offspring tolerance to OA (sticklebacks, North Sea, Schade et al. 2014), which may ameliorate these effects. The extent to which such effects operate in commercially important species in the Baltic-Skagerrak System is not yet known, however, and currently available data indicate that overall impacts of OA on commercially important fish stocks in the region will likely be negative.

Many of these findings come from coastal habitats, in which diurnal and seasonal pH variations can greatly exceed the shifts expected under OA (western Baltic, Saderne et al. 2013; Kattegat, Carstensen et al. 2018). However, the biological effects of such diurnal pH fluctuations in combination with OA have been investigated rarely (reviewed by Boyd et al. 2016). The only study to test these effects in the Baltic-Skagerrak System investigated responses of the euryhaline barnacle *Balanus improvisus* (Eriander et al. 2015). This study showed that diurnally fluctuating OA, mimicking future coastal seas, increased variance in growth more than tenfold, revealing strong “winner” and “loser” phenotypes that were not seen in conventional constant OA treatments, and which indicated the potential for adaptation to future OA. Similar differences in responses under constant *vs* fluctuating OA have been obtained in several other species (Boyd et al. 2016). Collectively, these findings indicate that tolerance to OA in situ may be far more variable than previously thought, and that projecting future impacts of OA from data obtained in experiments using constant OA treatments may be unreliable.

## Interactive effects of ocean acidification: Species-level responses

OA does not operate in isolation, and interactive and indirect effects (Box 4) are common. Additional drivers such as warming, freshening, and nutrient input are changing simultaneously in the Baltic-Skagerrak System (Meier 2015), leading to concurrent shifts in eutrophication and hypoxia (Conley et al. 2009, 2011), which interact with the effects of OA. Identifying the consequences of these multiple simultaneous drivers is a complex task (Boyd et al. 2018), yet many investigators have begun to address these in the Baltic-Skagerrak System (Table 1). This section focuses on the species-level effects of these interactions. Indirect effects, which operate via the food-web are considered in the following section.

**Table:** 

**Box 4** Direct, indirect, and interactive effects	
Anthropogenic drivers (including OA) rarely operate in isolation. Combinations of driver may have additive, synergistic, or antagonistic effects (Todgham and Stillman 2013). *Additive effects* are linear, and arise when the effects of multiple combined drivers equals the sum of their effects in isolation. In contrast, synergistic and antagonistic effects are non-linear and arise when the combined effects of multiple drivers is greater than (*synergistic*) or less than (*antagonistic*) the sum of those drivers in isolation (Todgham and Stillman 2013; Gunderson et al. 2016). Note that, these non-linear terms relate to the outcome of the interaction, not the effect itself: synergistic negative effects of temperature and OA on growth, for example, will create a greater *decrease* in growth than the sum of their single effects. Drivers also have direct, and/or indirect, effects on the focal organism or process. *Direct effects* (of single or multiple drivers) operate directly, such as the impacts of temperature on metabolic rate. *Indirect effects* operate through chains of interactions, e.g. decreased growth of a filter-feeder due to acidification-induced reduction in nutritional value of its phytoplankton food. Indirect effects typically operate through the food-web, and can be at least as great as direct effects (Alsterberg et al. 2013).	

Over the last 150 years, the Baltic has warmed by 0.4–1.5 °C (Gustafsson et al. 2012). Regional climate models project warming in this region will average + 2–3 °C by the end of this century (Meier 2015). This warming will have substantial direct impacts on marine organisms both independently (e.g. Boyd et al. 2013), and interactively with OA (Doney et al. 2012). Theoretical frameworks for the interaction between warming and acidification are well established (Pörtner and Farrell 2008), and recent work has confirmed this, showing that OA not only reduces the thermal tolerance window, but also can reduce peak performance at optimal temperature (larval cod, Kattegat, Dahlke et al. 2017), and modify intestinal physiology (adult cod, Kattegat, Hu et al. 2016). Other examples show, for example, reduced survival and growth under OA + warming (macroalgae, western Baltic, Al-Janabi et al. 2016b). Surprisingly, however, this pattern is far from uniform, and several investigations have found small, or no, interactive effects of OA + warming (e.g. crustaceans, western Baltic, Pansch et al. 2012; cod, western Baltic, Frommel et al. 2013), or additive effects that averaged the responses seen under each driver independently (e.g. copepods, Gulf of Finland, Vehmaa et al. 2012). Consequently, it is not possible to generalise the biological effects of this interaction (Table 1).

Interactions between OA and salinity are fundamental to the biogeochemistry of OA in the Baltic-Skagerrak System (Box 2, 3). Climate models project freshening by ~ 2 (≈ 10–20%) in the Belt-Seas around Denmark, and by 0.5–1 (≈ 15–30%) in the Bothnian Bay by 2100 (Christiansen et al. 2015; Meier 2015). This will drive species distributions further “out” toward the North Sea (Viitasalo et al. 2015). The likely combined biological effects of this freshening with OA in the Baltic-Skagerrak System have received relatively little attention to date. Available data indicate increased sensitivity to OA in low-salinity populations (crustaceans, Gulf of Bothnia, Wood et al. 2016), potential for freshening to counteract the positive effects of OA on reproductive success (*Fucus*, Baltic, Serraõ et al. Serrao et al. 1999; Graiff et al. 2017), and no substantive impacts of OA + freshening on microplankton community structure (Baltic Proper, Wulff et al. 2018; Table 1). This important research area requires further experimental investigation before patterns in responses to this interaction can be identified.

The increased precipitation that is projected to lead to freshening will also flush more inorganic and organic nutrients from land into the Baltic-Skagerrak System. The effects of this nutrient enrichment are well understood: it stimulates planktonic primary production, which contributes to increased sedimentation of organic matter to the sea-floor, where it decomposes, reducing O_2_ concentrations (creating hypoxia) and simultaneously generating CO_2_ and acidification (Conley et al. 2011; Meier et al. 2011; Meier 2015; Schneider et al. 2015; Box 3). The effects of hypoxia on marine organisms can be strong, and include behavioural changes, reduced performance, and loss of fitness (Gray et al. 2002), as well as reduced benthic productivity and biodiversity (Weigel et al. 2015). The combined effects of OA and hypoxia are now attracting renewed interest from the scientific community, and recent work in the Baltic-Skagerrak System has shown that OA + hypoxia can cause immune suppression in crustaceans (Skagerrak, Hernroth et al. 2015). Other workers, however, have found no effects of OA + hypoxia (*Mytilus*, southern Baltic, Jakubowska and Normant 2015), and even strong positive responses have been reported (*Macoma,* Gulf of Finland, Jansson et al. 2015; Table 1). The long-term prevalence of hypoxia in the Baltic-Skagerrak System (Conley et al. 2011) has almost certainly led to acclimation and adaption in many species, however, the extent to which this has also generated tolerance to OA is an exciting prospect that remains to be investigated.

## Indirect effects of ocean acidification: Ecosystem responses

Indirect effects of OA—which operate through shifts in competition and trophic interactions—are common in experiments that expose ecological communities to elevated pCO_2_. The indirect effects of OA on plankton communities vary among systems, but are typically small and subtle. In bacterioplankton, indirect effects were generally small (Baltic Proper, Lindh et al. 2013; western Baltic, Bergen et al. 2016; Table 1), although one study reported substantial shifts in microbial community composition correlated with shifts in phytoplankton community structure (Gulf of Finland, Hornick et al. 2017). Similar, subtle responses were observed in other studies of OA on community composition of phytoplankton (western Baltic, Sommer et al. 2015; Baltic Proper, Paul et al. 2016a, b), although stronger effects were seen in sub-dominant and rare species of phytoplankton (Sommer et al. 2015). These small indirect effects of OA can depend on nutrient availability: when nutrients were not limiting, either through experimental addition or in situ remineralisation, increased pCO_2_ shifted phytoplankton community composition towards much smaller picophytoplankton (Spitzbergen, Schulz et al. 2013; Kattegat, Bach et al. 2016). This, in turn, indirectly stimulated the microbial loop, which reduced subsequent energy flow to zooplankton and fish (North Sea, Endres et al. 2014; Gulf of Finland, Bermúdez et al. 2016). At an ecosystem level, indirect effects among planktonic species generally lead to small or no effects of OA on community composition (Skagerrak, Horn et al. 2016; Algueró-Muñiz et al. 2017; Langer et al. 2017) and genetic composition (Skagerrak, Langer et al. 2017), although some studies have reported reduced zooplankton biomass (Skagerrak, Taucher et al. 2017), and reduced community respiration (Gulf of Finland, Spilling et al. 2016). Importantly, OA impacts on fatty acid (FA) content of phytoplankton led to radical shifts in FA content, and reduced reproductive output in grazing copepods, indicating the potential for strong OA effects to propagate up the food-web (western Baltic, Rossoll et al. 2012). Field observations of plankton communities also indicate warming-induced shifts toward smaller size composition, with consequent reduction in energy availability to planktivorous fish (Suikkanen et al. 2013). Given that shifts in copepod abundance, size-spectrum, and nutritional quality are likely to cascade to higher trophic levels—and potentially influence regime shifts between cod-dominated and sprat/herring-dominated states in the Baltic (Casini et al. 2009)—there is a clear need to better understand the consequences of indirect and interactive effects of OA in plankton communities of the Baltic-Skagerrak System.

In benthic ecosystems, indirect effects of OA have been reported to be at least as great as direct effects. For example, mesocosm experiments in the Skagerrak showed OA alone had few or no direct or indirect effects, but the combination of OA with warming strongly influenced community composition and productivity (Eklöf et al. 2012, 2015; Alsterberg et al. 2013). These results were similar, but not identical, to those seen in response to warming alone (Alsterberg et al. 2013). Indirect trophic effects were also important: warming together with the loss of a keystone consumer strongly promoted the growth of ephemeral algae (Alsterberg et al. 2013), and had additive positive effects on functional diversity of associated fauna (Eklöf et al. 2015), such that overall indirect effects were at least as strong as direct effects (Alsterberg et al. 2013). Dense stands of benthic macrophytes can also generate substantial diurnal and seasonal pH fluctuations (globally, Hofmann et al. 2011; western Baltic, Saderne et al. 2013), which indirectly impact associated flora and fauna. For example, Baltic macroalgal and seagrass communities generate sufficiently large fluctuations in pH that they can provide temporal refuges from OA stress for calcifying species such as the blue mussel, *Mytilus edulis* (Wahl et al. 2018). The potential for dense stands of benthic macrophytes to condition the water, and thereby mitigate the impacts of OA throughout the coastal photic zone is clearly considerable (Saderne et al. 2013; Wahl et al. 2018), and warrants further investigation.

Indirect effects of OA also impact benthic-pelagic coupling, which plays a key ecological role throughout the Baltic-Skagerrak System. Sedimentation of planktonic primary production transfers food-energy to the benthos, which—in sufficiently high quantities—can increase food supply to benthic macrofauna, thereby increasing their resilience to OA (western Baltic, Thomsen and Melzner 2010; Melzner et al. 2011; Thomsen et al. 2013; Pansch et al. 2014; Stapp et al. 2017). However, the observation that OA reduces the nutritional value of phytoplankton for copepods (western Baltic, Rossoll et al. 2012), indicates that indirect effects of OA in combination with other drivers are likely to negatively influence the food value of particulates reaching benthic filter-feeders. The extent to which this reduction in food quality is offset by any OA-induced increases in total planktonic primary production (Kremp et al. 2012; Eberlein et al. 2017) remains unclear. Benthic macrofauna in the Baltic-Sakgerrak System are nonetheless projected to be negatively impacted by other, non-OA, drivers such as eutrophication, hypoxia, warming, and freshening (Weigel et al. 2015), which may have cascading effects throughout the ecosystem (Bergström et al. 2015; Viitasalo et al. 2015; Vuorinen et al. 2015), potentially driving regime shifts similar to those seen in the pelagic (Casini et al. 2009).

Modelling shows that controlling eutrophication and fishing pressure is central to managing Baltic ecosystems—not least because responses are non-linear (Niiranen et al. 2013). To date, however, these models have not included direct or indirect effects of OA. In regions where ecosystem management models have included the effects of OA, additive, synergistic, and antagonistic effects of OA with other drivers have been found (e.g. SE Australia, Griffith et al. 2011; US West coast, Kaplan et al. 2010). Including the effects of OA in ecosystem modelling of the Baltic-Skagerrak System is an important, and currently lacking, step that will permit assessment of ecosystem sensitivity to OA. Doing this calls for: (i) improved empirical knowledge/data on the effects of OA on the physiology and demography of key species, and on ecosystem responses; and (ii) modelling that includes the effects of OA at individual, population, and ecosystem levels (e.g. Koenigstein et al. 2016).

In addition to the drivers discussed above, other anthropogenic drivers have specific interactions with OA that influence the chemistry—and potentially biological responses—of the Baltic-Skagerrak System. Among these, shipping contributes directly to OA through the release not only of CO_2_ but also of the acidic gases SO_x_ and NO_x_ (Omstedt et al. 2015), which dissolve in seawater to create sulphuric and nitric acids. Methods to reduce SO_X_ emissions to the atmosphere include exhaust-gas scrubber systems that absorb the SO_x_ in a counterflow of seawater spray, creating a saline solution of sulphuric acid. Open-loop scrubbers that lack effluent treatment systems release this acid direct to surface waters, causing further acidification. Although the effects of such release are small on a basin scale (Turner et al. 2018), in heavily trafficked areas the effects of releasing acidic scrubber effluent on local seawater pH can be as large as those of anthropogenic ocean acidification (Stips et al. 2016). The biological effects of additional effluent components such as organic microparticles are poorly known.

Warming is also causing marine species distributions to move poleward (so-called “climate tracking”; Pinsky et al. 2013; Hiddink et al. 2015; Molinos et al. 2016). This is projected to increase species turnover (gain of new species, loss of established species) in the coming decades (Molinos et al. 2016). Invasive species are already established throughout the Baltic-Skagerrak System (Hiddink and Coleby 2012; Katsanevakis et al. 2014), with both negative and positive impacts on marine biogeochemistry and ecosystems (reviewed by Katsanevakis et al. 2014) that can interact with OA (Box 3). Although there is strong uncertainty about the timing of further invasions, the establishment of invasive species—especially those tolerant of low pH—seems likely. At the time of writing, no published studies have investigated the effects of OA on warming-driven range shifts and ecosystem structure and function in the Baltic-Skagerrak System.

## The role of biodiversity, evolution, and resilience

The impacts of OA and other anthropogenic drivers on marine ecosystems depend not only on the factors and processes considered above. Biodiversity (both genetic variation within species, as well as between species [i.e. richness]), functional diversity (the diversity of ecological functions within an ecosystem), and evolutionary capacity of the component species, are all central to determining the resilience of an ecosystem to disturbance and change (Doney et al. 2012; Loreau and Mazancourt 2013; Gamfeldt et al. 2015; Lefcheck et al. 2015), and hence to OA.

At a within-species-level, anthropogenic pressures are reducing population sizes and genetic diversity (Harley et al. 2006). In smaller populations, stochastic factors such as genetic drift can restrict the ability of populations to adapt to local conditions (Polechová and Barton 2015). Within-species genetic diversity in the Skagerrak and Kattegat is substantially greater than in populations of the same species in the Baltic (Johannesson and André 2006), and consequently we might expect the adaptive capacity of Baltic populations to OA to be less than that in the relatively diverse populations of the Skagerrak and Kattegatt. At present, there are no geographically comprehensive data with which we can test this prediction. Nonetheless, it is clear that some Baltic populations are strongly tolerant to extreme levels of OA (e.g. mussels, western Baltic, Thomsen et al. 2017), and that Baltic populations have adapted rapidly in the past to drivers such as salinity change (Pereyra et al. 2009; and see Johannesson et al. 2011). Experimental laboratory work has also shown that Baltic species can adapt rapidly to high *p*CO_2_ conditions (e.g. Lohbeck et al. 2012; Stapp et al. 2017). Although the greater complexity of the natural environment may lead to different outcomes (Bach et al. 2018), available evidence nonetheless suggests there is substantial adaptive capacity in Baltic populations.

At the level of ecological communities, loss of species reduces ecosystem functioning, leading to loss of productivity and greater sensitivity to disturbance (Campbell et al. 2011; Cardinale et al. 2012; Worm et al. 2006; Duffy et al. 2016). Thus, biodiversity is critical to the long-term sustainability of ecosystem functions in the face of environmental change (Loreau and Mazancourt, 2013; Oliver et al. 2015). Examples from marine ecosystems include genetic diversity enhancing the resilience of Baltic seagrass beds (Reusch et al. 2005), and species diversity enhancing the resilience of the California Current pelagic ecosystem (Lindegren et al. 2016). The extent to which resilience to OA is enhanced by biodiversity is less well understood, although work in Swedish seagrass beds indicates that OA + warming can have positive indirect impacts (Alsterberg et al. 2013), and warming (but not OA) reduces the insurance effect of biodiversity (Eklöf et al. 2012). Conversely, investigations of the effects of OA on genetic diversity of plankton communities in large mesocosms found no substantive impacts (Skagerrak, Langer et al. 2017). Unfortunately, comparable data from the Baltic Proper are not available. Nonetheless, it seems likely that the reduced biodiversity observed within and among species in the Baltic Sea will decrease ecosystem resilience to OA and other interacting drivers, rendering the Baltic particularly vulnerable.

## Consequences for ecosystem services

The impacts of OA on marine ecosystems are likely to have pervasive consequences. The world’s coastal oceans, including the Baltic-Skagerrak System, generate numerous ecosystem services that are important for human welfare (Millennium Ecosystem Assessment 2005; TEEB 2010; Hattam et al. 2015). Identifying and forecasting the effects of OA on these services in the Baltic-Skagerrak System is, however, a challenging task for several reasons. First, attributing any given ecosystem change to an individual driver, such as OA, is rarely straightforward because multiple interacting drivers simultaneously cause ecosystem change. Shifts in ecosystem services driven by overfishing, eutrophication, habitat destruction, and OA, for example, have their origins in distal phenomena such as urbanisation, global consumption patterns, trade, etc. (Norström et al. 2017; Österblom et al. 2017). This is exemplified in the Baltic-Skagerrak System by eutrophication-driven ecosystem degradation, which has contributed to declines in marine ecosystem goods and services (Rönnbäck et al. 2007; Österblom et al. 2007). Second, these different impacts often have synergistic (reinforcing) or antagonistic (mitigating) effects on each other (Box 4). Ecosystem disturbances operating over long periods can result in small gradual changes in ecosystem structure and functions, yet can sometimes (and especially when disturbances are strong and synergistic) cause rapid shifts that change ecosystem structure, functions, and services (Scheffer et al. 2001; Österblom et al. 2007; Casini et al. 2009). Conversely, negative impacts on extant fisheries may be offset by positive impacts on new, alternate, fishery species (similar to the effects of ocean warming on fisheries catch, Cheung et al. 2010, but see Cheung et al. 2016), although such trade-offs have yet to be demonstrated for OA. Third, the diversity of ecosystem services implies that OA is likely to affect them in different ways. Among the provisioning ecosystem services alone there are multiple examples, such as the production of fish, which can be impacted by the negative effects of OA on coastal fish species (Baltic herring, Franke and Clemmesen 2011; Kattegatt cod, Stiasny et al. 2016); the negative effects of OA on aquaculture (e.g. mussel and oyster growth in the USA, Barton et al. 2012); and the possibility of indirect effects of OA on forage fish in distant waters that that are used for aquaculture feeds. Similar OA-driven shifts can also arise in supporting and cultural ecosystem services through impacts on coastal towns reliant on the fishing industry. Finally, people benefiting from these services may value them in very different ways and will be affected differently depending on their own vulnerability and whether or not replacement services exist.

The potential risks for economic losses can be illustrated by looking at just one key provisioning service, the Swedish fishery sector. In 2016, Swedish fisheries were conservatively estimated to be worth ~ SEK 7.5 billion (recreational = SEK 6.2 billion, SCB 2018; industrial = SEK 1.3 billion, HAV 2014), equivalent to ~ € 730 million. Including Swedish aquaculture (SEK 487 million; SCB 2017), pushes this total to ~ € 780 million. Of course, not all of this value will be lost as a result of OA impacts (and much aquaculture value is in freshwaters), but as outlined earlier, commercially valuable fish species in the Baltic-Skagerrak System respond negatively to OA, and therefore this key provisioning service could incur substantial financial costs. Currently, however, know too little to make valid quantitative assessments of the likely social and economic risks and vulnerabilities arising from biological responses to OA (Falkenberg and Tubb 2017). In order to make these assessments, we need to meet the relevant requirements (Hilmi et al. 2013): (i) accurate determination of the fraction of GNP that depends on provisioning ecosystem services such as fishing and aquaculture, and cultural services such as coastal tourism, that depend on OA sensitive ecosystems; (ii) identifying likely shifts in species composition, and hence food value, of seafood under OA; (iii) projecting changes in human populations dependent on the coastal zone; (iv) determining the vulnerability and sensitivity of these coastal populations to environmental change and assessing their capacity to adapt.

## Conclusions

The Baltic-Skagerrak System is characterised by seasonal and diurnal cycles of temperature, primary production, and decomposition, which create strong cycles of pH variation. Interactions with local biogeochemistry and anthropogenic drivers modify these fluctuations, making the detection of decadal pH trends from field observations difficult. However, oceanographic models for the Baltic Proper that incorporate the marine carbonate system project increasing seasonal pH variability and clear long-term (multi-decadal) reductions in mean, and minimum, pH. The results of these models are consistent with projected global trends (Fig. 6; Omstedt et al. 2009, 2012).

The responses of marine organisms in the Baltic-Skagerrak System to levels of ocean acidification (OA) consistent with these trends (pCO_2_ ≤ 1300 µatm; pH decline ≤ 0.4 units) vary widely from strongly positive, through neutral, to strongly negative. In general, however, we detected a pattern in which species at higher trophic levels tended respond more negatively to OA. Negative responses to OA were more common in macrobenthos and fish than in plankton. Responses to OA were strongly modified by several factors, most prevalent of which was nutritional status (nutrients for primary producers, food for consumers), such that more nutrients/food led to greater resilience to OA. Investigations of the combined effects of OA with warming, freshening, nutrients, or hypoxia frequently showed strong interactions that overwhelmed (warming), or materially changed (hypoxia), responses in comparison to those seen under OA only. In many instances interactive effects of OA + other drivers were small or not detected, and responses to other drivers (notably warming) were greater than those to OA.

Responses to OA also vary widely among taxonomic levels: species-level responses were generally stronger (both positive and negative) than responses of communities or ecosystems—indicating the importance of indirect interactions in the ecosystem, which can mediate OA effects on individual taxa. Overall ecosystem (mesocosm) level effects of OA were generally small, although there was evidence for substantial shifts in genetic composition of some plankton communities.

## Outlook and research priorities

The findings summarised here represent substantial advances in OA research in the Baltic-Skagerrak System in the last 6 years (*cf* Havenhand 2012). Nonetheless, our understanding of the interactive and indirect effects of OA remains constrained by our limited knowledge of several key processes, and we know woefully little about how Baltic-Skagerrak species and ecosystems will respond to OA under diurnally, and seasonally, fluctuating cycles (Eriander et al. 2015; Boyd et al. 2016). We echo the sentiments of recent reviews that have emphasised the importance of moving from single drivers, single species, and short timescales, to multiple drivers, ecological communities, and evolutionary responses (e.g. Wernberg et al. 2012; Riebesell and Gattuso 2015; Boyd et al. 2018). In particular, we recommend that future research in the Baltic-Skagerrak System region focus on:

(i) How biogeochemical processes combine to impact the development of OA in brackish coastal seas, and the role of nutrient enrichment and eutrophication; in particular, develop models to project future levels of important drivers under different forcing.
(ii) Quantifying the additive, synergistic (reinforcing), and antagonistic (mediating) responses of keystone, and ecologically dominant, species to OA in combination with other important anthropogenic drivers (notably warming, eutrophication, hypoxia, biodiversity, and fishing).
(iii) Determining the extent of phenotypic plasticity and adaptive capacity of key Baltic-Skagerrak species over multiple generations in response to OA, in combination with other important drivers.
(iv) Quantifying the effects of intra- and interspecific biodiversity on ecosystem responses and resilience to OA in combination with other key drivers; in particular the capacity for OA responses to cascade through the food-web.
(v) Determining the effects of diurnal and seasonal environmental fluctuations, superimposed on OA and other key drivers, on responses of species and ecosystems
(vi) Developing holistic ecosystem modelling frameworks that incorporate the effects of OA with those of other drivers at large spatial scales
(vii) Evaluating the impacts these processes will have on socially and economically important ecosystem services.

## Footnotes

1 Since the onset of industrialisation, global atmospheric CO_2_ levels have increased by ~ 45% (from ~ 270 to today’s 400 µatm), causing seawater pH to decrease by about 0.1 units. pH is projected to decrease by another 0.3 units by 2100. Because of the logarithmic nature of the pH scale, these changes equate to increases of ~ 30% and ~ 100% (respectively) in the hydrogen ion content of seawater (Box 1).

2 The practical salinity scale is dimensionless and therefore we refer to salinity throughout this document simply as a number.

3 Connell et al. 2018 note the duality of CO_2_ as both a resources and a stressor. Therefore, as far as practicable, we follow the DPSIR definition of “drivers” (“*…complex phenomena governing the direction of ecosystem change, which could be of human and natural origin*”), of Oesterwind et al. (2016).
